# Supplementary material for: Speciation in a biodiversity hotspot: Phylogenetic relationships, species delimitation, and divergence times of Patagonian ground frogs from the Eupsophus roseus group (Alsodidae)
Source: PLoS One. 2018 Dec 13;13(12):e0204968. doi: 10.1371/journal.pone.0204968 (PMC6292574; doi:10.1371/journal.pone.0204968)
Supplement: S1 Table — Coordinates, sample size (N), corresponding species according to Frost [35] and map number from Fig 1 are indicated. Species used as outgroup are also listed (gray cells). (DOC) [file pone.0204968.s001.doc]

**S1 Table. Sampling locations of *Eupsophus* species**. Coordinates, sample size (N), corresponding species according to Frost [35] and map number from Fig 1 are indicated. Species used as outgroup are also listed (gray cells).

| **Nº Map** | **Location** | **Latitude** | **Longitude** | **N** | **Species** |
| --- | --- | --- | --- | --- | --- |
| 1 | Los Queules (QE) | -35.99278 | -72.52583 | 12 | *E. roseus* |
| 2 | Nahuelbuta (NA) | -37.78861 | -72.99222 | 9 | *E. roseus* |
| 3 | Contulmo (CO) | -38.01253 | -73.18716 | 7 | *E. roseus* |
| 4 | Villarrica (VI) | -39.26528 | -71.89222 | 16 | *E. roseus* |
| 5 | Pocura (PO) | -39.47584 | -72.03990 | 9 | *E. roseus* |
| 6 | Lago Pellaifa (LP) | -39.61037 | -71.98545 | 8 | *E. roseus* |
| 7 | Huifco, torre 21 (HF) | -39.61083 | -72.89889 | 3 | *E. roseus* |
| 8 | San Pablo de Tregua (ST) | -39.61322 | -72.09214 | 2 | *E. roseus* |
| 9 | San Martin (SM) | -39.62879 | -73.18702 | 1 | *E. roseus* |
| 10 | Fundo Santa Maria (FS) | -39.66683 | -73.18123 | 7 | *E. roseus* |
| 11 | Mafil, Torre 41 (MA) | -39.68059 | -72.92016 | 2 | *E. roseus* |
| 12 | Llancahue (LA) | -39.83943 | -73.13014 | 11 | *E. roseus* |
| 13 | Chamil (CH) | -40.01070 | -73.10796 | 1 | *E. roseus* |
| 14 | Paillaco, Torre 140 (PAI) | -40.02942 | -72.87912 | 1 | *E. roseus* |
| 15 | Pichirropulli (PI) | -40.15894 | -72.88596 | 4 | *E. roseus* |
| 16 | Los Mañios (LM) | -40.33256 | -72.32542 | 1 | *E. roseus* |
| 17 | Isla Mocha (IM) | -38.37732 | -73.89002 | 5 | *E. insularis* |
| 18 | Mehuin (ME) | -39.41167 | -73.11198 | 7 | *E. migueli* |
| 19 | Chanchan (CC) | -39.58014 | -73.22376 | 6 | *E. migueli* |
| 20 | Oncol (ON) | -39.69845 | -73.32725 | 1 | *E. migueli* |
| 21 | Reumen, torre 117 (RH) | -39.95463 | -72.89952 | 3 | *E. calcaratus* |
| 22 | Reumen, torre 119 (RT) | -39.95556 | -72.88750 | 1 | *E. calcaratus* |
| 23 | Reumen (RE) | -39.96778 | -72.90111 | 2 | *E. calcaratus* |
| 24 | Cordillera Pelada (CP) | -40.14028 | -73.41778 | 2 | *E. calcaratus* |
| 25 | Bahía Mansa (BM) | -40.56306 | -73.73167 | 2 | *E. calcaratus* |
| 26 | Antillanca (AN) | -40.66889 | -72.16028 | 2 | *E. calcaratus* |
| 27 | Sarao (SA) | -41.16417 | -73.72722 | 10 | *E. calcaratus* |
| 28 | Alerce Andino (AA) | -41.58028 | -72.54083 | 2 | *E. calcaratus* |
| 29 | Puntra (PT) | -42.10389 | -73.87000 | 2 | *E. calcaratus* |
| 30 | Pumalín (PU) | -42.61639 | -72.47806 | 2 | *E. calcaratus* |
| 31 | El Amarillo (EA) | -42.89444 | -72.46833 | 3 | *E. calcaratus* |
| 32 | Yaldad(YA) | -43.10528 | -73.69556 | 2 | *E. calcaratus* |
| 33 | Isla Guafo (IG) | -43.60278 | -74.72083 | 2 | *E. calcaratus* |
| 34 | Marín Balmaceda (MB) | -43.78389 | -72.96389 | 1 | *E. calcaratus* |
| 35 | La Junta (LJ) | -43.93944 | -72.36361 | 1 | *E. calcaratus* |
| 36 | Lago Verde(LV) | -44.23472 | -71.84083 | 2 | *E. calcaratus* |
| 37 | Queulat (QU) | -44.37694 | -72.54139 | 2 | *E. calcaratus* |
| 38 | Pto Aguirre(PG) | -45.15694 | -73.52111 | 1 | *E. calcaratus* |
| 39 | Isla Vergara (IV) | -45.18778 | -73.52833 | 1 | *E. calcaratus* |
| 40 | Pto Aysén (PA) | -45.42361 | -72.68778 | 2 | *E. calcaratus* |
| 41 | Bahía Murta (BU) | -46.45083 | -72.66778 | 2 | *E. calcaratus* |
| 42 | Caleta Tortel (CT) | -47.79500 | -73.53000 | 2 | *E. calcaratus* |
| 43 | Lago Quetru (QT) | -48.09111 | -73.11750 | 2 | *E. calcaratus* |
| 12 | Llancahue (LA) | -39.83943 | -73.13014 | 1 | *E. vertebralis* |
| 19 | Mehuin (ME) | -39.41167 | -73.11198 | 1 | *E. vertebralis* |
| 22 | Reumén (RE) | -39.95463 | -72.89952 | 1 | *E. vertebralis* |
| 44 | Huillinco (HI) | -42.67316 | -74.00801 | 1 | *E. emiliopugini* |
| 45 | Pucatrihue (PC) | -40.57641 | -73.70304 | 1 | *E. emiliopugini* |
| 19 | Chanchan (CC) | -39.58014 | -73.22376 | 1 | *A. norae* |
