# Supplementary material for: Speciation in a biodiversity hotspot: Phylogenetic relationships, species delimitation, and divergence times of Patagonian ground frogs from the Eupsophus roseus group (Alsodidae)
Source: PLoS One. 2018 Dec 13;13(12):e0204968. doi: 10.1371/journal.pone.0204968 (PMC6292574; doi:10.1371/journal.pone.0204968)
Supplement: S2 Table — Conservative (C), variable (V), informative (I) and total sites for each marker are indicated. Partitioning schemes and nucleotide substitution models were determined using Partitionfinder, version 2.1.1 [56]. (DOC) [file pone.0204968.s002.doc]

**S2 Table. Sites characterization, partitioning schemes, and nucleotide substitution models for sequences used in this study**. Conservative (C), variable (V), informative (I) and total sites for each marker are indicated. Partitioning schemes and nucleotide substitution models were determined using Partitionfinder, version 2.1.1 [58].

| **Sequences** | **C** | **V** | **I** | **Total sites** | **Subset Partitions** | **Best Model** |
| --- | --- | --- | --- | --- | --- | --- |
| D-loop | 246 | 288 | 221 | 540 | D-loop | HKY+G |
| Cyt *b* | 378 | 233 | 202 | 611 | Codon position 1 from Cyt *b* and COI | K80+I+G |
| COI | 419 | 229 | 206 | 648 | Codon position 2 from Cyt *b* and COI | F81+I |
| CRYBA1 | 184 | 31 | 19 | 216 | Codon position 3 from Cyt *b* and COI | GTR+G |
| POMC | 484 | 77 | 52 | 561 | POMC and CRYBA1 | HKY+I+G |
