# Supplementary material for: Speciation in a biodiversity hotspot: Phylogenetic relationships, species delimitation, and divergence times of Patagonian ground frogs from the Eupsophus roseus group (Alsodidae)
Source: PLoS One. 2018 Dec 13;13(12):e0204968. doi: 10.1371/journal.pone.0204968 (PMC6292574; doi:10.1371/journal.pone.0204968)
Supplement: S3 Table — Mean of all the Ctax values obtained involving a given approach (Mean Ctax) and total number of species supported by each approach (sp.) is indicated. Species delimitation approaches: Bayesian General Mixed Yule Coalescent model (bGMYC), multi-rate Poisson Tree Processes (mPTP), Tree Estimation using Maximum likelihood, (STEM), Bayesian Species Delimitation (BPP), Multi-locus Species Delimitation using a Trinomial Distribution Model (Tr2), and Bayes factor delimitation (BFD). (DOC) [file pone.0204968.s003.doc]

**S3 Table. Taxonomic index of congruence (*Ctax*) calculated for each pair of approaches.** Mean of all the *Ctax* values obtained involving a given approach (Mean C*tax*) and total number of species supported by each approach (sp.) is indicated. Species delimitation approaches: Bayesian General Mixed Yule Coalescent model (bGMYC), multi-rate Poisson Tree Processes (mPTP), Tree Estimation using Maximum likelihood, (STEM), Bayesian Species Delimitation (BPP), Multi-locus Species Delimitation using a Trinomial Distribution Model (Tr2), and Bayes factor delimitation (BFD).

|  | **C*tax*** | | | | | | **Mean C*tax*** | **sp.** |
| --- | --- | --- | --- | --- | --- | --- | --- | --- |
|  | **bGMYC** | **mPTP** | **STEM** | **BPP** | **Tr2** | **BFD** |
| **bGMYC** | - |  |  |  |  |  | 0.27 | 40 |
| **mPTP** | 0.50 | - |  |  |  |  | 0.44 | 20 |
| **STEM** | 0.20 | 0.40 | - |  |  |  | 0.63 | 8 |
| **BPP** | 0.23 | 0.45 | 0.89 | - |  |  | 0.69 | 9 |
| **Tr2** | 0.23 | 0.45 | 0.89 | 1.00 | - |  | 0.69 | 9 |
| **BFD** | 0.20 | 0.40 | 0.78 | 0.89 | 0.89 | - | 0.63 | 8 |
