# Supplementary material for: Speciation in a biodiversity hotspot: Phylogenetic relationships, species delimitation, and divergence times of Patagonian ground frogs from the Eupsophus roseus group (Alsodidae)
Source: PLoS One. 2018 Dec 13;13(12):e0204968. doi: 10.1371/journal.pone.0204968 (PMC6292574; doi:10.1371/journal.pone.0204968)
Supplement: S4 Table — Species delimitation scenarios for Eupsophus species are indicated in Fig 4A. Species number (sp.), Log-likelihood of the species tree (−lnL), number of parameters (k), AIC, AIC difference (Δi), the relative likelihood of model given the data (L), and the model probabilities (wi) are indicated. Note the proximity between–lnL from scenario 11 and 12. (DOC) [file pone.0204968.s004.doc]

**S4 Table. Likelihood scores and Akaike’s information criterion (AIC) results for STEM analysis (see Carstens and Dewey [18]).** Species delimitation scenarios for *Eupsophus* species are indicated in Fig 4A. Species number (sp.), Log-likelihood of the species tree (−lnL), number of parameters (k), AIC, AIC difference (*Δi*), the relative likelihood of model given the data (*L*), and the model probabilities (*w_i_*) are indicated. Note the proximity between –lnL from scenario 11 and 12.

| **Scenarios** | **sp.** | **-lnL** | ***k*** | **AIC** | ***Δi*** | ***L*(ModelData*)*** | ***w_i_*** |
| --- | --- | --- | --- | --- | --- | --- | --- |
| 11 | 8 | -113727.8336 | 9 | 227473.667 | 0.000 | 1.000 | 0.657 |
| 12 | 9 | -113727.9731 | 10 | 227475.946 | 2.279 | 0.320 | 0.210 |
| 8 | 7 | -113730.7049 | 8 | 227477.410 | 3.743 | 0.154 | 0.101 |
| 10 | 8 | -113730.8444 | 9 | 227479.689 | 6.022 | 0.049 | 0.032 |
| 13 | 10 | -113755.2107 | 11 | 227532.421 | 58.754 | 0.000 | 0.000 |
| 7 | 7 | -115093.3335 | 8 | 230202.667 | 2729.000 | 0.000 | 0.000 |
| 9 | 8 | -115093.4730 | 9 | 230204.946 | 2731.279 | 0.000 | 0.000 |
| 5 | 6 | -115096.2047 | 7 | 230206.409 | 2732.742 | 0.000 | 0.000 |
| 6 | 6 | -116056.3823 | 7 | 232126.765 | 4653.097 | 0.000 | 0.000 |
| 4 | 5 | -116059.2511 | 6 | 232130.502 | 4656.835 | 0.000 | 0.000 |
| 2 | 4 | -116094.0395 | 5 | 232198.079 | 4724.412 | 0.000 | 0.000 |
| 3 | 4 | -116169.5463 | 5 | 232349.093 | 4875.425 | 0.000 | 0.000 |
| 1 | 3 | -116173.4853 | 4 | 232354.971 | 4881.303 | 0.000 | 0.000 |
