# Supplementary material for: Speciation in a biodiversity hotspot: Phylogenetic relationships, species delimitation, and divergence times of Patagonian ground frogs from the Eupsophus roseus group (Alsodidae)
Source: PLoS One. 2018 Dec 13;13(12):e0204968. doi: 10.1371/journal.pone.0204968 (PMC6292574; doi:10.1371/journal.pone.0204968)
Supplement: S5 Table — Marginal likelihood (MLE) and Bayes factor estimates for species delimitation scenarios indicated in Fig 4A. Species number (sp.) as well as values using path (PS) and stepping-stone (SS) sampling are indicated. (DOC) [file pone.0204968.s005.doc]

**S5 Table. Bayes factor delimitation results.** Marginal likelihood (MLE) and Bayes factor estimates for species delimitation scenarios indicated in Fig 4A. Species number (sp.) and values using path (PS) and stepping-stone (SS) sampling are indicated.

| **Scenario** | **sp.** | **MLE (PS)** | **Bayes Factor (PS)** | **MLE (SS)** | **Bayes Factor (SS)** |
| --- | --- | --- | --- | --- | --- |
| 1 | 3 | -13333.057 | -13332.940 | 18.718 | 17.810 |
| 2 | 4 | -13327.934 | -13328.191 | 8.471 | 8.313 |
| 3 | 4 | -13340.928 | -13341.003 | 34.459 | 33.937 |
| 4 | 5 | -13329.581 | -13329.937 | 11.766 | 11.804 |
| 5 | 6 | -13326.720 | -13327.138 | 6.043 | 6.207 |
| 6 | 6 | -13331.583 | -13331.997 | 15.769 | 15.925 |
| 7 | 7 | -13329.242 | -13329.100 | 11.087 | 10.130 |
| 8 | 7 | -13325.929 | -13326.053 | 4.461 | 4.037 |
| 9 | 8 | -13326.100 | -13325.985 | 4.805 | 3.900 |
| 10 | 8 | -13323.698 | -13324.035 | 0.000 | 0.000 |
| 11 | 8 | -13329.336 | -13329.306 | 11.276 | 10.542 |
| 12 | 9 | -13326.166 | -13326.586 | 4.935 | 5.103 |
| 13 | 10 | -13327.780 | -13328.420 | 8.164 | 8.770 |
