# Supplementary figures and images for: Speciation in a biodiversity hotspot: Phylogenetic relationships, species delimitation, and divergence times of Patagonian ground frogs from the Eupsophus roseus group (Alsodidae)
Source: PLoS One. 2018 Dec 13;13(12):e0204968. doi: 10.1371/journal.pone.0204968 (PMC6292574; doi:10.1371/journal.pone.0204968)

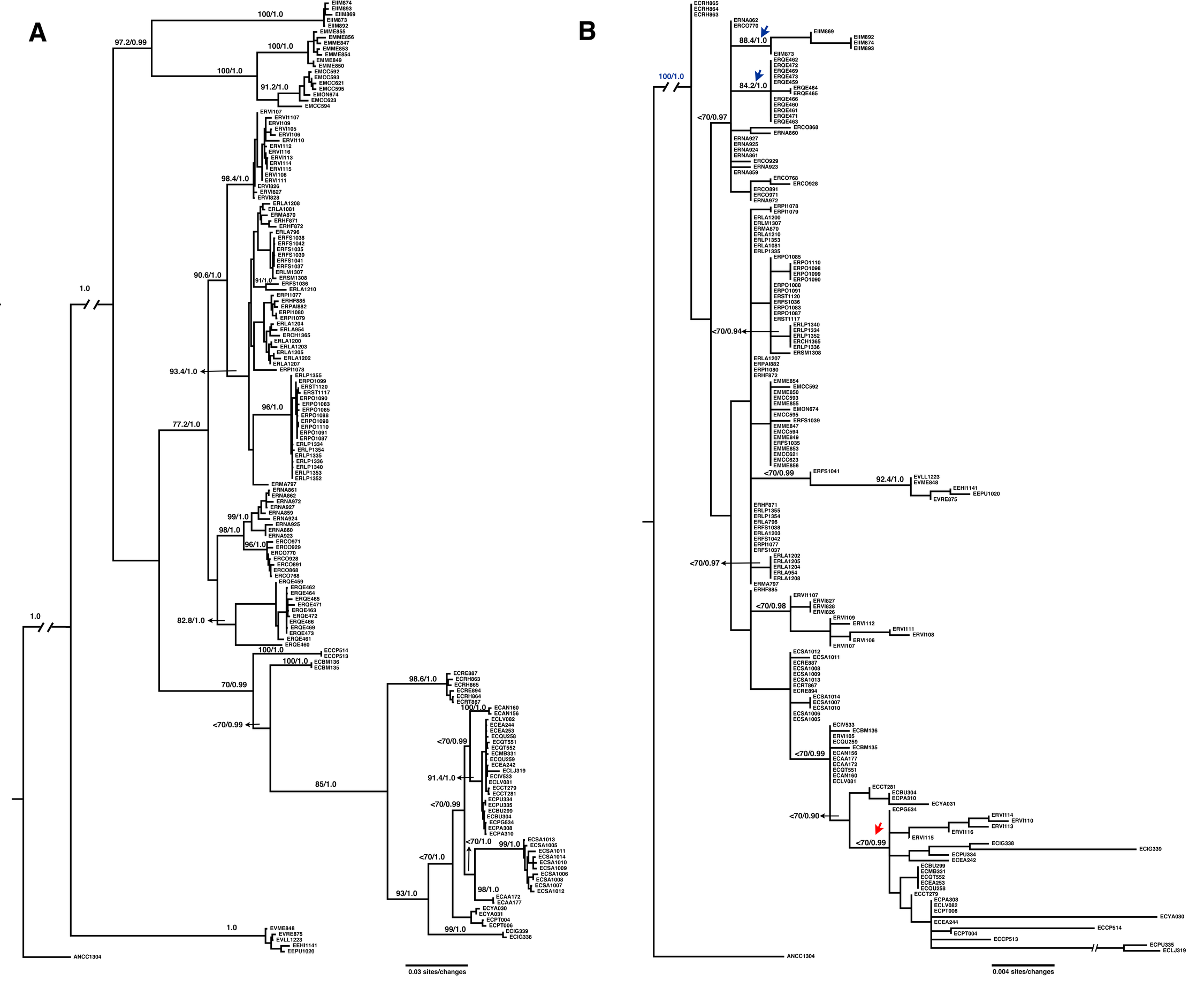

Supplement: S1 Fig — Maximum likelihood trees using A) mitochondrial (D-loop, Cytb, and COI) and B) nuclear (POMC and CRYBA1) sequences. Bayesian analyses recovered similar topologies in both cases. Support values (bootstrap and posterior probabilities) are shown above branches. Blue arrows indicate lineages 1 and 8 shown in Fig 2, red arrow indicates a clade supported only in Bayesian analyses. (TIF) [file pone.0204968.s006.tif]
