# Supplementary material for: Speciation in a biodiversity hotspot: Phylogenetic relationships, species delimitation, and divergence times of Patagonian ground frogs from the Eupsophus roseus group (Alsodidae)
Source: PLoS One. 2018 Dec 13;13(12):e0204968. doi: 10.1371/journal.pone.0204968 (PMC6292574; doi:10.1371/journal.pone.0204968)
Supplement: S1 File — Automatic Barcode Gap Discovery (ABGD) results using A) COI and B) concatenated (D-loop, Cytb, COI, POMC and CRYBA1) datasets. (DOCX) [file pone.0204968.s007.docx]

**S1 File. Automatic Barcode Gap Discovery (ABGD) results using A) *COI* and B) concatenated (*D-loop*, *Cytb*, *COI*, *POMC* and *CRYBA1*) datasets.**

1. **COI data set results**

Kimura distance Partition 1 : found 27 groups (prior maximal distance P= 0.001000)
Partition 2 : found 9 groups (prior maximal distance P= 0.001847)
Partition 3 : found 9 groups (prior maximal distance P= 0.003411)
Partition 4 : found 9 groups (prior maximal distance P= 0.006300)
Partition 5 : found 5 groups (prior maximal distance P= 0.011635)
Partition 6 : found 3 groups (prior maximal distance P= 0.021488)


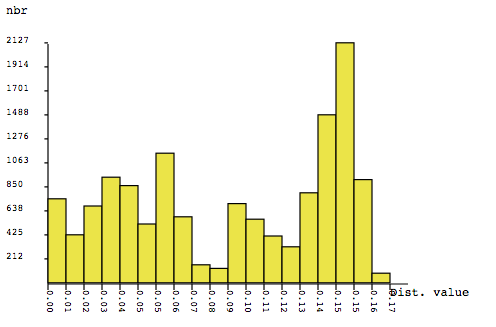


1

2

1

2


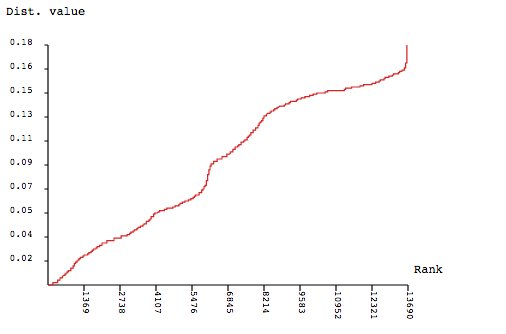


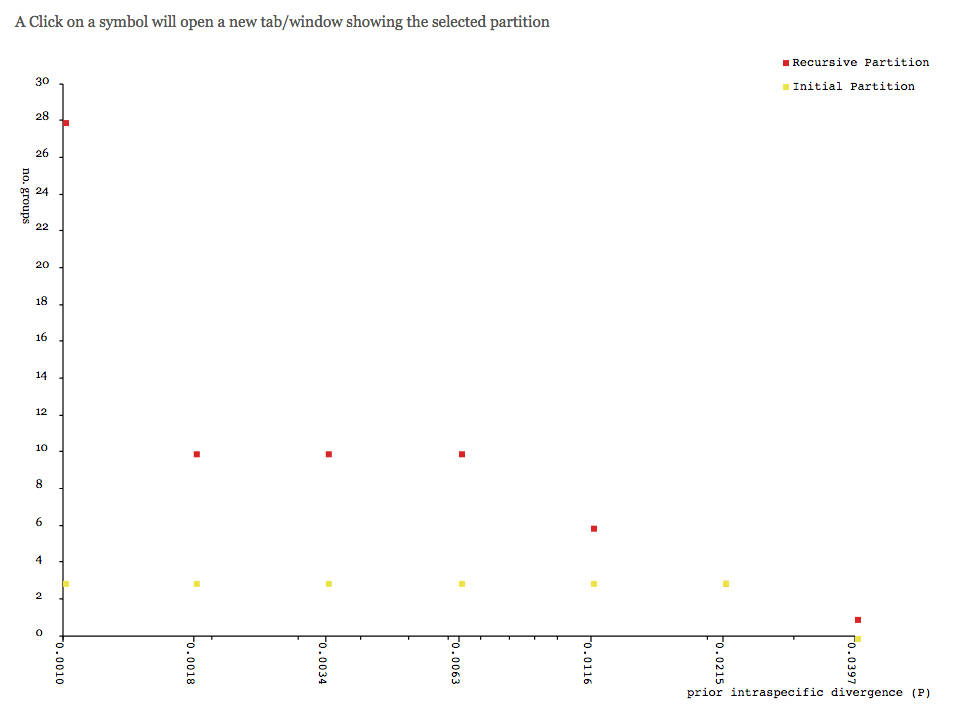


2

1

1

2

Partition with prior maximal distance P=6.30e-03
Distance K80 Kimura MinSlope=1.500000
Download (left click and save) or see below the tree file corresponding to this partition: click [here](http://wwwabi.snv.jussieu.fr/public/abgd/temp/30163.126990986/groupe4.tree)

**Group[ 1 ] n: 112 ;**id: ERLP1335 ERLP1334 ERLP1336 ERLP1340 ERLP1353 ERLP1352 ERPO1087 ERPO1090 ERPO1098 ERPO1099 ERPO1110 ERST1120 ERPO1091 ERPO1085 ERPO1088 ERLP1355 ERLP1354 ERPO1083 ERST1117 ERMA797 ERHF871 ERHF872 ERLA1081 ERLA1208 ERFS1035 ERFS1037 ERFS1041 ERFS1042 ERFS1038 ERFS1039 ERLA796 ERLM1307 ERSM1308 ERMA870 ERPI1078 ECBM135 ECBM136 ECCP514 ECCP512 ECCP513 ERLA1205 ERLA1207 ERLA1202 ERCH1365 ERHF885 ERPAI882 ERPI1079 ERPI1080 ERPI1077 ERLA1204 ERLA954 ERLA1200 ERLA1203 ERFS1036 ERLA1210 ESPVI112 ESPVI1107 ESPVI111 ESPVI116 ESPVI109 ESPVI108 ESPVI105 ESPVI113 ESPVI114 ESPVI115 ESPVI106 EspVI828 EspVI826 ESPVI110 EspVI827 ESPVI107 ECCO768 ECCO770 ECCO868 ECCO891 ECCO928 ECCO971 ECCO929 ENNA859 ENNA924 ENNA862 ENNA972 ENNA861 ENNA927 ENNA860 ENNA923 ENNA925 ESQE459 ESQE461 ESQE462 ESQE463 ESQE466 ESQE469 ESQE471 ESQE473 ESQE465 ESQE464 ESQE472 ESQE460 EACH595 EACC621 EACH593 EACH592 EAON674 EACC623 EACH594 EMME853 EMME855 EMME854 EMME847 EMME856 EMME849 EMME850
**Group[ 2 ] n: 5 ;**id: EIIM869 EIIM873 EIIM892 EIIM893 EIIM874
**Group[ 3 ] n: 4 ;**id: ECAA172 ECAA177 ECAN156 ECAN160
**Group[ 4 ] n: 6 ;**id: ECRT867 ECRE894 ECRH864 ECRH865 ECRE887 ECRH863
**Group[ 5 ] n: 10 ;**id: ECSA1005 ECSA1013 ECSA1012 ECSA1008 ECSA1011 ECSA1006 ECSA1007 ECSA1010 ECSA1014 ECSA1009
**Group[ 6 ] n: 2 ;**id: ECIG338 ECIG339
**Group[ 7 ] n: 2 ;**id: ECPT006 ECPT004
**Group[ 8 ] n: 9 ;**id: ECBU299 ECBU304 ECPA308 ECPA310 ECPG534 ECPU334 ECPU335 ECYA030 ECYA031
**Group[ 9 ] n: 14 ;**id: ECQT551 ECQT552 ECCT279 ECCT281 ECQU259 ECQU258 ECEA244 ECEA253 ECEA242 ECMB331 ECIV533 ECLV081 ECLV082 ECLJ319

Partition with prior maximal distance P=1.16e-02
Distance K80 Kimura MinSlope=1.500000
Download (left click and save) or see below the tree file corresponding to this partition: click [here](http://wwwabi.snv.jussieu.fr/public/abgd/temp/15833.1612466290/groupe5.tree)

Partition with prior maximal distance P=1.16e-02
Distance K80 Kimura MinSlope=1.500000
Download (left click and save) or see below the tree file corresponding to this partition: click [here](http://wwwabi.snv.jussieu.fr/public/abgd/temp/15833.1612466290/groupe5.tree)

**Group[ 1 ] n: 113 ;**id: ERLP1335 ERLP1334 ERLP1336 ERLP1340 ERLP1353 ERLP1352 ERPO1087 ERPO1090 ERPO1098 ERPO1099 ERPO1110 ERST1120 ERPO1091 ERPO1085 ERPO1088 ERLP1355 ERLP1354 ERPO1083 ERST1117 ERMA797 ERHF871 ERHF872 ERLA1081 ERLA1208 ERFS1035 ERFS1037 ERFS1041 ERFS1042 ERFS1038 ERFS1039 ERLA796 ERLM1307 ERSM1308 ERMA870 ERPI1078 ECBM135 ECBM136 ECCP514 ECCP512 ECCP513 ERLA1205 ERLA1207 ERLA1202 ERCH1365 ERHF885 ERPAI882 ERPI1079 ERPI1080 ERPI1077 ERLA1204 ERLA954 ERLA1200 ERLA1203 ERFS1036 ERLA1210 ESPVI112 ESPVI1107 ESPVI111 ESPVI116 ESPVI109 ESPVI108 ESPVI105 ESPVI113 ESPVI114 ESPVI115 ESPVI106 EspVI828 EspVI826 ESPVI110 EspVI827 ESPVI107 ECCO768 ECCO770 ECCO868 ECCO891 ECCO928 ECCO971 ECCO929 ENNA859 ENNA924 ENNA862 ENNA972 ENNA861 ENNA927 ENNA860 ENNA923 ENNA925 ESQE459 ESQE461 ESQE462 ESQE463 ESQE466 ESQE469 ESQE471 ESQE473 ESQE465 ESQE464 ESQE472 ESQE460 EACH595 EACC621 EACH593 EACH592 EAON674 EACC623 EACH594 EMME853 EMME855 EMME854 EMME847 EMME856 EMME849 EMME850
**Group[ 2 ] n: 5 ;**id: EIIM869 EIIM873 EIIM892 EIIM893 EIIM874
**Group[ 3 ] n: 31 ;**id: ECAA172 ECAA177 ECAN156 ECAN160 ECIG338 ECIG339 ECPT006 ECPT004 ECBU299 ECBU304 ECPA308 ECPA310 ECPG534 ECPU334 ECPU335 ECYA030 ECYA031 ECQT551 ECQT552 ECCT279 ECCT281 ECQU259 ECQU258 ECEA244 ECEA253 ECEA242 ECMB331 ECIV533 ECLV081 ECLV082 ECLJ319
**Group[ 4 ] n: 6 ;**id: ECRT867 ECRE894 ECRH864 ECRH865 ECRE887 ECRH863
**Group[ 5 ] n: 10 ;**id: ECSA1005 ECSA1013 ECSA1012 ECSA1008 ECSA1011 ECSA1006 ECSA1007 ECSA1010 ECSA1014 ECSA1009

1. **Concatenated data set results**

Kimura distance Partition 1 : found 13 groups (prior maximal distance P= 0.001000)
Partition 2 : found 13 groups (prior maximal distance P= 0.001847)
Partition 3 : found 10 groups (prior maximal distance P= 0.003411)
Partition 4 : found 7 groups (prior maximal distance P= 0.006300)
Partition 5 : found 4 groups (prior maximal distance P= 0.011635)


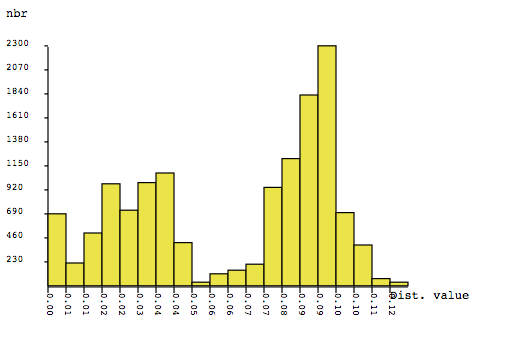


1

2


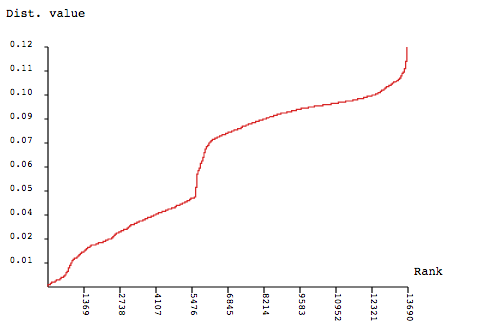


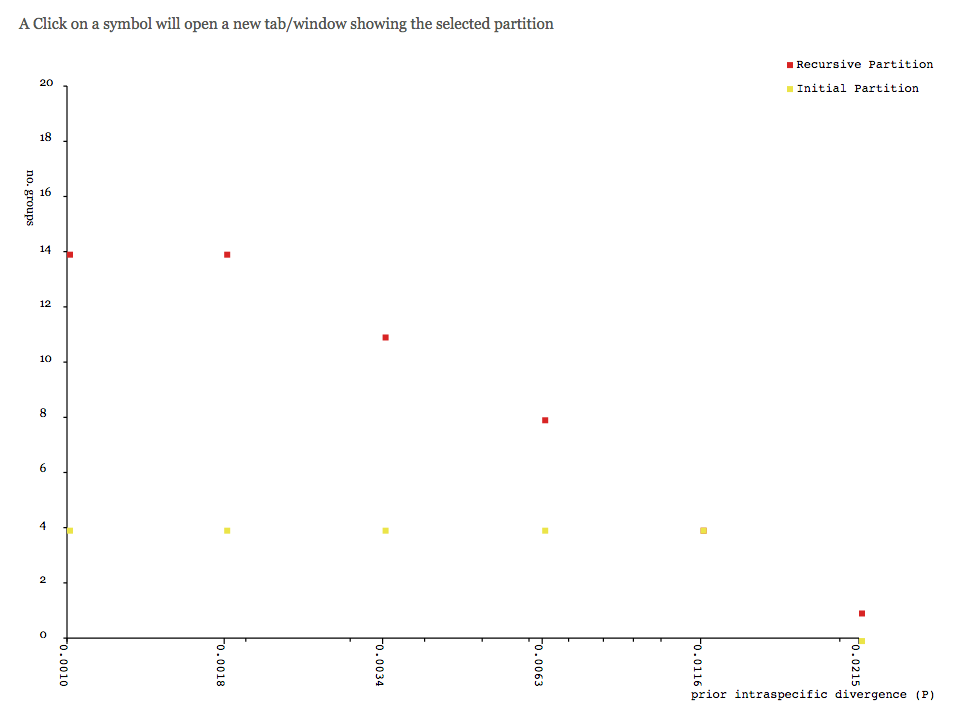


1

2

**1)** Partition with prior maximal distance P=6.30e-03
Distance K80 Kimura MinSlope=1.500000
Download (left click and save) or see below the tree file corresponding to this partition: click [here](http://wwwabi.snv.jussieu.fr/public/abgd/temp/29096.90262758/groupe4.tree)

**Group[ 1 ] n: 99 ;**id: ECCP514 ECCP513 ECBM135 ECBM136 ERST1117 ERST1120 ERPO1110 ERPO1099 ERPO1098 ERPO1091 ERPO1090 ERPO1088 ERPO1087 ERLP1355 ERLP1354 ERLP1353 ERLP1352 ERLP1340 ERLP1335 ERLP1334 ERLP1336 ERPO1083 ERPO1085 ERHF872 ERHF871 ERMA797 ERFS1035 ERFS1037 ERFS1038 ERFS1039 ERFS1041 ERLM1307 ERSM1308 ERFS1042 ERLA796 ERPI1078 ERLA1081 ERLA1208 ERMA870 ERHF885 ERPI1079 ERPI1077 ERPI1080 ERPAI882 ERLA1200 ERLA1207 ERLA1202 ERLA1205 ERLA1203 ERLA1204 ERLA954 ERCH1365 ERFS1036 ERRE8 ERLA1210 ECCO868 ECCO891 ECCO768 ECCO928 ECCO929 ECCO770 ECCO971 ENNA927 ENNA972 ENNA859 ENNA861 ENNA862 ENNA924 ENNA860 ENNA923 ENNA925 ESQE464 ESQE466 ESQE469 ESQE473 ESQE462 ESQE461 ESQE463 ESQE472 ESQE465 ESQE459 ESQE471 ESQE460 ESPVI105 ESPVI106 ESPVI1107 ESPVI108 ESPVI111 ESPVI107 ESPVI109 ESPVI110 ESPVI113 ESPVI114 ESPVI115 ESPVI116 ESPVI112 EspVI827 EspVI828 EspVI826
**Group[ 2 ] n: 7 ;**id: EACC621 EACH593 EACH595 EACH592 EAON674 EACC623 EACH594
**Group[ 3 ] n: 5 ;**id: EIIM873 EIIM892 EIIM893 EIIM869 EIIM874
**Group[ 4 ] n: 39 ;**id: ECAA172 ECAA177 ECSA1005 ECSA1011 ECSA1006 ECSA1008 ECSA1010 ECSA1014 ECSA1009 ECSA1007 ECSA1013 ECSA1012 ECAN156 ECAN160 ECBU304 ECEA244 ECEA253 ECLV081 ECPA308 ECPT004 ECPT006 ECQT552 ECQU259 ECYA030 ECYA031 ECQU258 ECBU299 ECEA242 ECPU335 ECIV533 ECPG534 ECPU334 ECCT279 ECCT281 ECPA310 ECQT551 ECLV082 ECMB331 ECLJ319
**Group[ 5 ] n: 2 ;**id: ECIG338 ECIG339
**Group[ 6 ] n: 6 ;**id: ECRE887 ECRH865 ECRE894 ECRH864 ECRT867 ECRH863
**Group[ 7 ] n: 7 ;**id: EMME847 EMME856 EMME855 EMME849 EMME850 EMME853 EMME854

**2)** Partition with prior maximal distance P=1.16e-02
Distance K80 Kimura MinSlope=1.500000
Download (left click and save) or see below the tree file corresponding to this partition: click [here](http://wwwabi.snv.jussieu.fr/public/abgd/temp/29096.90262758/groupe.init.5.tree)

**Group[ 1 ] n: 98 ;**id: ECCP514 ECCP513 ECBM135 ECBM136 ERST1117 ERST1120 ERPO1110 ERPO1099 ERPO1098 ERPO1091 ERPO1090 ERPO1088 ERPO1087 ERLP1355 ERLP1354 ERLP1353 ERLP1352 ERLP1340 ERLP1335 ERLP1334 ERLP1336 ERPO1083 ERPO1085 ERHF872 ERHF871 ERMA797 ERFS1035 ERFS1037 ERFS1038 ERFS1039 ERFS1041 ERLM1307 ERSM1308 ERFS1042 ERLA796 ERPI1078 ERLA1081 ERLA1208 ERMA870 ERHF885 ERPI1079 ERPI1077 ERPI1080 ERPAI882 ERLA1200 ERLA1207 ERLA1202 ERLA1205 ERLA1203 ERLA1204 ERLA954 ERCH1365 ERFS1036 ERLA1210 ECCO868 ECCO891 ECCO768 ECCO928 ECCO929 ECCO770 ECCO971 ENNA927 ENNA972 ENNA859 ENNA861 ENNA862 ENNA924 ENNA860 ENNA923 ENNA925 ESQE464 ESQE466 ESQE469 ESQE473 ESQE462 ESQE461 ESQE463 ESQE472 ESQE465 ESQE459 ESQE471 ESQE460 ESPVI105 ESPVI106 ESPVI1107 ESPVI108 ESPVI111 ESPVI107 ESPVI109 ESPVI110 ESPVI113 ESPVI114 ESPVI115 ESPVI116 ESPVI112 EspVI827 EspVI828 EspVI826
**Group[ 2 ] n: 47 ;**id: ECAA172 ECAA177 ECSA1005 ECSA1011 ECSA1006 ECSA1008 ECSA1010 ECSA1014 ECSA1009 ECSA1007 ECSA1013 ECSA1012 ECAN156 ECAN160 ECBU304 ECEA244 ECEA253 ECLV081 ECPA308 ECPT004 ECPT006 ECQT552 ECQU259 ECYA030 ECYA031 ECQU258 ECBU299 ECEA242 ECPU335 ECIV533 ECPG534 ECPU334 ECCT279 ECCT281 ECPA310 ECQT551 ECLV082 ECMB331 ECLJ319 ECIG338 ECIG339 ECRE887 ECRH865 ECRE894 ECRH864 ECRT867 ECRH863
**Group[ 3 ] n: 14 ;**id: EACC621 EACH593 EACH595 EACH592 EAON674 EACC623 EACH594 EMME847 EMME856 EMME855 EMME849 EMME850 EMME853 EMME854
**Group[ 4 ] n: 5 ;**id: EIIM873 EIIM892 EIIM893 EIIM869 EIIM874
